# High mobility group B1 impairs hepatocyte regeneration in acetaminophen hepatotoxicity

**DOI:** 10.1186/1471-230X-12-45

**Published:** 2012-05-08

**Authors:** Runkuan Yang, Shutian Zhang, Antonella Cotoia, Niku Oksala, Shengtao Zhu, Jyrki Tenhunen

**Affiliations:** 1 Department of Intensive Care Medicine, University of Tampere Medical School, Tampere 33014, Finland; 2 Department of Critical Care Medicine, University of Pittsburgh Medical School, 3550 Terrace Street, Pittsburgh, PA 15261, USA; 3 Department of Gastroenterology, Friendship Hospital, Capital Medical School, Beijing, China; 4 Center of Laboratory Medicine, Tampere University Hospital and Surgery, University of Tampere Medical School, Tampere 33014, Finland; 5 Department of Surgical Sciences/Anaesthesiology and Intensive Care, University of Uppsala Medical School, Uppsala 75185, Sweden

## Abstract

**Background:**

Acetaminophen (APAP) overdose induces massive hepatocyte necrosis. Necrotic tissue releases high mobility group B1 (HMGB1), and HMGB1 contributes to liver injury. Even though blockade of HMGB1 does not protect against APAP-induced acute liver injury (ALI) at 9 h time point, the later time points are not studied and the role of HMGB1 in APAP overdose is unknown, it is possible that neutralization of HMGB1 might improve hepatocyte regeneration. This study aims to test whether blockade of HMGB1 improves hepatocyte regeneration after APAP overdose.

**Methods:**

Male C57BL/6 mice were treated with a single dose of APAP (350 mg/kg). 2 hrs after APAP administration, the APAP challenged mice were randomized to receive treatment with either anti-HMGB1 antibody (400 μg per dose) or non-immune (sham) IgG every 24 hours for a total of 2 doses.

**Results:**

24 hrs after APAP injection, anti-HMGB1 therapy instead of sham IgG therapy significantly improved hepatocyte regeneration microscopically; 48 hrs after APAP challenge, the sham IgG treated mice showed 14.6% hepatic necrosis; in contrast, blockade of HMGB1 significantly decreased serum transaminases (ALT and AST), markedly reduced the number of hepatic inflammatory cells infiltration and restored liver structure to nearly normal; this beneficial effect was associated with enhanced hepatic NF-κB DNA binding and increased the expression of cyclin D1, two important factors related to hepatocyte regeneration.

**Conclusion:**

HMGB1 impairs hepatocyte regeneration after APAP overdose; Blockade of HMGB1 enhances liver recovery and may present a novel therapy to treat APAP overdose.

## Background

Acetaminophen hepatotoxicity is the leading cause of drug-induced acute liver failure (ALF) in the United States and other industrialized nations [1]. Massive necrosis is the dominant feature of APAP –induced ALI [2-6] and necrotic tissue passively releases HMGB1 [7-9], an important late inflammatory mediator that was well studied in sepsis [10], and HMGB1 contributes to liver injury [11,12]; this indicates that HMGB1 might play an important role in the pathogenesis of APAP hepatotoxicity. Although blockade of HMGB1 in an APAP-induced ALI model does not protect against liver injury at 9 h point, inflammation is reduced as seen by myeloperoxidase (MPO) activity in total liver extract [9], however, the later time points are not studied and the role of HMGB1 in APAP overdose is still not known. It is possible that neutralization of HMGB1 might improve hepatocyte regeneration in APAP toxicity.

Based on these observations, we hypothesized that HMGB1 impairs hepatocyte regeneration after APAP overdose and treated APAP challenged mice with anti-HMGB1 neutralizing antibody or non-immune IgG for 24 or 48 hours.

## Methods

### Materials

All chemicals were purchased from Sigma-Aldrich Chemical Co. (St. Louis, MO, USA) unless otherwise noted. Polyclonal antibodies against HMGB1 were raised in rabbits (Cocalico Biologicals, Reamstown, PA, USA), and titers were determined by immunoblotting as previously described [13]. Anti–HMGB1 antibodies were affinity-purified by using cyanogen bromide–activated Sepharose beads following standard procedures. Neutralizing activity of anti-HMGB1 was confirmed in HMGB1-stimulated macrophage cultures by assay of TNF-α release. In the presence of anti-HMGB1 antibody, neutralizing antibody was defined as inhibiting > 80% of HMGB1-induced TNF release. Sham IgG antibodies were purified from non-immunized rabbit IgG.

### Ethical considerations

This research protocol complied with the regulations regarding the care and use of experimental animals published by the National Institutes of Health and was approved by the Institutional Animal Use and Care Committee of the University of Tampere Medical School. Male C57BL/6 mice weighing 20–25 g (University of Kuopio animal care center, Kuopio, Finland) were used in this study. The animals were maintained at the University of Kuopio Animal Research Center with a 12-hour light–dark cycle and free access to standard laboratory food and water. The animals were fasted over night prior to the experiments.

### Animal experiments

In the first experiment, 10 mice were randomized into the APAP group and the control group (n = 5 for each group). 5 mice in the APAP group were i.p. injected with a single sub lethal dose of APAP (300 mg/kg dissolved in 1 mL sterile saline) and 5 mice in the control group were injected with same volume of saline not containing APAP. 24 hrs after APAP injection, the animals in each group were anesthetized with sodium pentobarbital (90 mg/kg i.p.) and blood was aspirated from the heart to measure serum HMGB1 by western blot.

In the second experiment, ALI was induced by a single dose of APAP (350 mg/kg dissolved in 1 mL sterile saline) administered by i.p. injection. 14 APAP challenged mice were then randomized into the anti-HMGB1 group (n = 6) and the sham IgG group (n = 8). 6 mice injected with saline not containing APAP served as the control group. The animals in the anti-HMGB1 group were given 1 dose of anti-HMGB1 antibody (400 μg per dose dissolved in 0.5 mL sterile saline) 2 hrs after APAP injection. The same amount of sham IgG and saline were given to the sham IgG group or the control group at equivalent time points. 24 hrs after APAP injection, all surviving mice in each group were anesthetized with sodium pentobarbital (90 mg/kg i.p.), serum was collected to measure ALT, AST and HMGB1 and the left lobe of the liver was stored in 10% formalin for pathology (HE staining).

In the third experiment, ALI was induced the same as above described. APAP injected mice were then randomized into the anti-HMGB1 group (n = 10) and the sham IgG group (n = 11). 6 mice injected with saline not containing APAP served as the control group. The animals in the anti-HMGB1 group were given 2 doses of anti-HMGB1 antibody (400 μg per dose dissolved in 0.5 mL sterile saline): the first dose was given 2 hrs after APAP injection, the second dose were given 24 hrs after the first dose of anti-HMGB1 antibody.

The same amount of sham IgG and saline were given to the sham IgG group or the control group at equivalent time points. 48 hrs after APAP injection, all surviving mice in each group were anesthetized with sodium pentobarbital (90 mg/kg i.p.), and the following procedures were performed: 1) blood was aspirated from the heart to measure serum ALT and AST; 2) the left lobe of the liver was stored in 10% formalin for pathology (HE staining); 3) the rest of the liver tissue was harvested and frozen for subsequent protein extractions.

In the fourth experiment, 3 separate groups of mice were used. ALI was induced the same as above described. The treatments remained the same as described in the second experiment except one more dose of anti-HMGB1 or sham IgG was given 48 h after the first dose and animals were sacrificed at 72 h time point. Except relatively smaller necrosis (8%) in the sham IgG group at 72 time point, the other parameters such as NF-κB and cyclin D1 at 72 h were comparable to the 48 h time point, therefore, this study focused on 24 h and 48 h time points.

### Serum HMGB1 measurements

HMGB1 levels were measured by western immunoblotting analysis as described [7]. In brief, serum samples (100 μL) were ultrafiltered with Centricon 100 (Millpore). The elute was fractionated by SDS/PAGE, transferred to nylon membranes (Bio-Rad), and probed with purified IgG from anti-HMGB1 antiserum (5 μg/ ml) for western blot analysis. Polyclonal anti-HMGB1 IgG was purified by using protein A agarose according to the manufacturer’s instructions (Pierce). Membranes were visualized with an Enhanced Chemiluminescence substrate (ECL, Amersham Pharmacia Biotech) and exposed to X-ray film according to the manufacturer’s instructions.

### Serum aminotransferase measurements

Serum levels of AST and ALT were measured at 37°C with a commercially available kit (Sigma Diagnostic).

### Myeloperoxidase assay for hepatic neutrophils infiltration

Neutrophils infiltration was measured at 48 hours by determining MPO activity in liver tissue homogenates as previously described [14] and was used as an index of neutrophils infiltration in all groups. The MPO levels were expressed as units per gram of tissue (U/g).

### Light histology studies

Formalin-fixed hepatic tissue was sectioned, stained with hematoxylin and eosin (H&E) and examined using light microscopy. Blind analysis was performed on all samples to determine the degree of lesion as previously described [15]. The percentage of necrosis was estimated by evaluating the number of microscopic fields with necrosis compared with the entire cross section. Inflammatory cell infiltration results were scored semi-quantitatively by averaging the number of inflammatory cells per microscopic field at a magnification of 200×. Five fields were evaluated per tissue sample; six animals in each group were examined at 24 h time point; six animals in the control group, nine animals in the sham IgG group and 10 animals in the anti-HMGB1 group were examined at 48 h.

### Western blot for cyclin D1 measurement

Liver protein was extracted as previously described [13]. Equivalent amounts of protein were boiled in sample buffer and subjected to 7.5% precast SDS-polyacrylamide gels (Bio-Rad) and transferred to nylon membranes. Membranes were then probed with a specific antibody against cyclin D1 (Cell signaling Technology, Lexington, KY) protein, visualized with an Enhanced Chemiluminescence substrate (ECL, Amersham Pharmacia Biotech) and exposed to X-ray film according to the manufacturer’s instructions.

### Assessment of NF-кB activation

NF-κB activation was determined by EMSA, as previously described [16]. The gels were dried and exposed to Biomax film (Kodak, Rochester, NY) at −70°C overnight with use of an intensifying screen.

### Hepatocyte proliferation (DNA synthesis)

Since at 24 h time point, the APAP challenged C57/BL6 mice only showed occasional hepatocyte nuclei labeled for 5-bromo-2-deoxyuridine (BrdU) [17], therefore, BrdU test was performed only at 48 h time point in this study. To evaluate hepatocyte regeneration, mice from the 48 h groups were administered BrdU (50-mg/ kg i.p. injection at 46 h time point) 2 hours before they were killed. Parraffin-embedded liver sections were prepared and processed for immunohistochemistry using BrdU *in-situ* staining kits from BD Pharmingen (San Jose, CA, USA) according to the manufacturer’s instructions. Digital images of 5 low-power fields from each liver were obtained in a random and blinded fashion, and the number of BrdU-labeled hepatocyte nuclei was counted. The average number of BrdU-positive hepatocytes in each animal was used for subsequent analysis.

### Statistics

Data are presented as means ± SEM. Continuous data were analyzed using student’s t-test or analysis of variance followed by Fisher’s LSD test. Probability levels less than 0.05 were considered significant (p < 0.05).

## Results

### 24 h time point serum HMGB1

**Figure 1:**
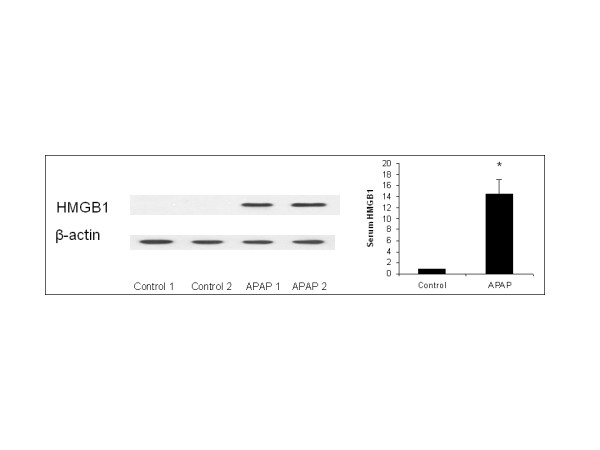
**Serum HMGB1 in APAP-induced ALI model.** ALI was induced in C57Bl/6 male mice with a single dose of APAP by intraperitoneal (i.p.) injection (300 mg/kg dissolved in 1 mL saline), each control animal was injected with 1 mL saline not containing APAP (n = 5 animals for each group). Western blot was performed by using serum obtained 24 hours after APAP injection. Results from 5 representative assays are shown (n = 5, each band stands for one serum sample from the control or APAP injected animals). Typical gels are depicted.

Western blot was performed to measure serum HMGB1 from the normal control and the APAP groups (n = 5 animals for each group, each band stands for one serum sample from the control or APAP groups). 24 hrs after APAP injection, the serum HMGB1 level in APAP challenged mice was significantly higher than that in the control group (Figure 1).

### Serum ALT and AST at 24 h and 48 h time points

**Figure 2:**
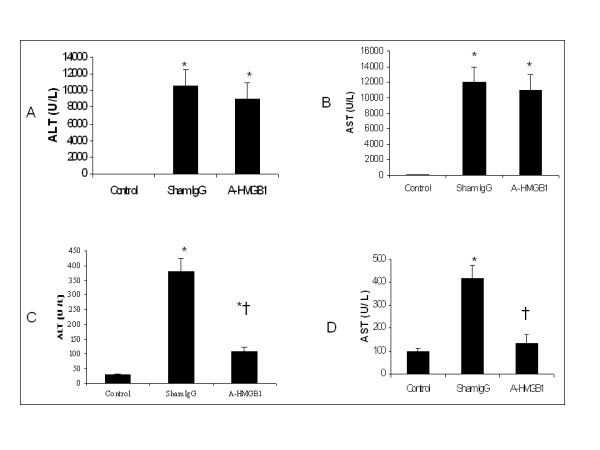
**Effect of treatment with anti-HMGB1 antibody or sham IgG on serum ALT/AST in acetaminophen (APAP)-induced acute liver injury (ALI).** ALI was induced in C57 BL/6 male mice with a single dose of APAP (350 mg/kg) by i.p. injection. 2 hours after APAP administration, the first dose of 400 μg of anti-HMGB1 antibody in 0.5 mL saline was i.p. injected into mice in the anti-HMGB1 group, the same amount of sham IgG or saline solution was given to the sham IgG and the control animals at the equivalent time points. The same dose was repeated 24 hrs after the first dose. Figure 2A, 2B: Serum ALT/AST was assessed 24 hrs after APAP injection from the anti-HMGB1 group, the sham IgG and the control group (n = 6 for each group). Figure 2C and 2D: 3 separate groups of mice were used. Serum ALT and AST were assessed at 48 h time point from the anti-HMGB1 group (n = 10), the sham IgG group (n = 9) and the control group (n = 6). Results are means ± SEM. * indicates p < 0.05 vs. control; † indicates p < 0.05 vs. sham IgG.

24 hrs after APAP injection, 2 mice from sham IgG group died, all mice in the anti-HMGB1 group and the control group survived. Anti-HMGB1 therapy did not statistically decrease serum ALT/AST than that in the sham IgG group (n = 6 for each group). 48 hrs after APAP challenge, the survival rate was 81.8% (9/11) in the sham IgG group and 100% (10/10) in the anti-HMGB1 therapy group; all mice survived in the control group. Repeatedly, serum ALT/AST decreased rapidly at 48 h time point as compared to 24 h time point. Compared to sham IgG treatment, blockade of HMGB1 significantly reduced serum concentrations of ALT and AST at 48 h time point (†indicates p < 0.05 vs. sham IgG) (Figure 2)

### Histopathology

**Figure 3:**
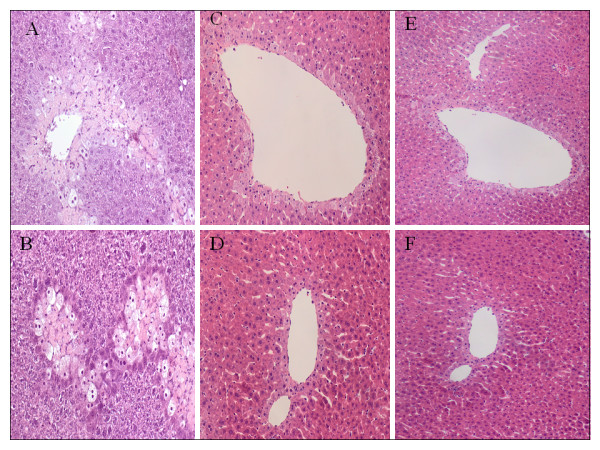
**Effect of treatment with anti-HMGB1 antibody or sham IgG on pathology in mice with ALI.** Hematoxylin-eosin staining was assessed 24 and 48 h after induction of ALI (or sham procedure). Method and treatment were the same as described in Figure 2 (n = 6 for each group at 24 h time point; at 48 h time point, n = 9 for the sham IgG group, n = 10 for the anti-HMGB1 group and n = 6 for the control group). Typical picture is shown. [A=sham IgG at 24 h (200x), B=anti-HMGB1 at 24 h (200x), C=sham IgG at 48 h (200x), D=anti-HMGB1 at 48 h (200x), E=sham IgG at 48 h (100x), F=anti-HMGB1 at 48 h (100x)].

24 hrs after APAP injection, microscopically, the most conspicuous feature was the evident hepatocyte regeneration in the anti-HMGB1 therapy group compared with sham IgG treated mice. In histological evaluation 48 hrs after ALI induction, compared to control animals, sham IgG treated mice demonstrated 14.6 ± 1.2% necrotic area and extensive infiltration of inflammatory cells (270 ± 40 per high power field, n = 9) in the centrilobular regions. Loss of cell boundaries and ballooning degeneration were also found around hepatic central vein in the sham IgG group. In contrast, anti-HMGB1 therapy significantly reduced the number of inflammatory cells (100 ± 35 per high power field, n = 10) and demonstrated 1.6 ± 0.3% necrosis around the central vein, the necrosis was not yet completely replaced by regeneration, even though the liver structure has restored to nearly normal (Figure 3).

### Liver tissue MPO level

At 48 h time point, the mean liver MPO activity value for the control group (n = 6) was 4.2 ± 0.29 U/g, this value increased to 5.64 ± 0.40 U/g in the sham IgG group (n = 9) and 4.90 ± 0.40 U/g in the anti-HMGB1 group (n = 10), no statistical difference between the sham IgG and anti-HMGB1 groups (p = 0.073, data were shown as Mean ± SEM).

### Hepatic NF-κB DNA binding

**Figure 4:**
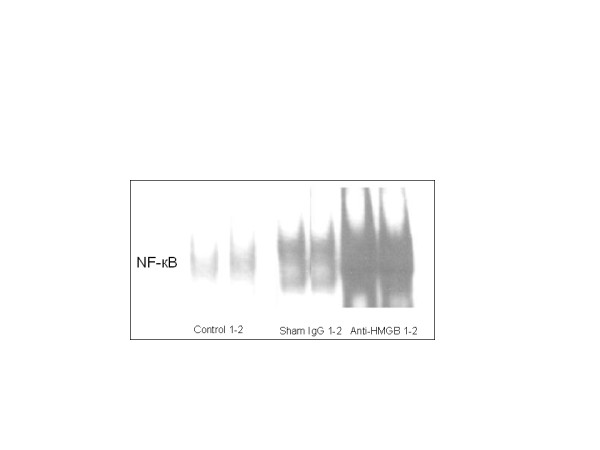
**Effect of treatment with anti-HMGB1 antibody or sham IgG on NF-кB DNA binding in nuclear extracts prepared from hepatic tissue samples from mice with ALI.** NF-кB DNA binding was assessed 48 h h after induction of ALI (or sham procedure). The figure depicts results from six representative assays. Typical gels are depicted.

NF-κB is a pleiotropic transcription factor whose activation has been linked to inflammatory and destructive processes, as well as initiation of regenerative programs in the injured liver. Blockade of HMGB1 protects against ischemia-reperfusion (I/R)-induced liver injury; this protection is associated with increased NF-κB DNA binding activity [12]. Therefore, we examined the impact of APAP on activation of NF-κB at 48 hours after APAP injection and tested the effect of HMGB1 blockade. There was a low basal level of NF- κB DNA binding in the hepatic tissue samples in the control group. In the sham IgG group, there was a marked increase in NF- κB DNA binding. Treatment of mice after APAP challenge with anti-HMGB1 neutralizing antibody clearly increased NF-κB DNA binding relative to the degree observed in mice treated with non-immune IgG (Figure 4). NF-κB DNA binding was not consistent at 24 h time point.

### Hepatic cyclin D1 expression

**Figure 5:**
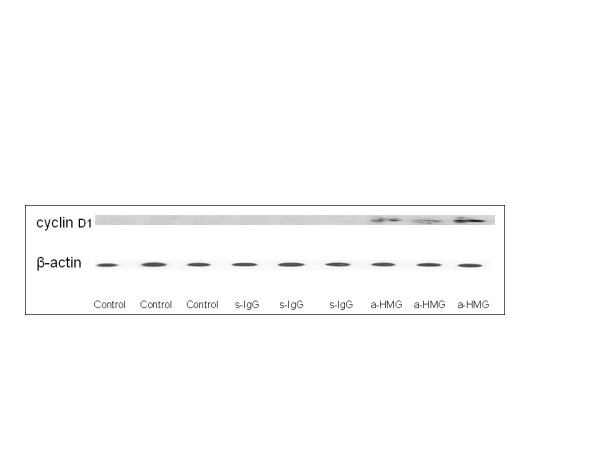
**Effect of treatment with anti-HMGB1 or sham IgG on the expression of cyclin D1 in the hepatic tissue.** Western blot was performed using hepatic extracts prepared from tissues obtained 48 hrs after APAP injection. The figure depicts results from six representative assays. Typical gels are depicted.

The induction of cyclin D1 is the most reliable marker for cell cycle (G1 phase) progression in hepatocytes [18] Western blot was performed using whole-cell extracts prepared from liver tissue to assess expression of cyclin D1 in mice subjected to ALI or the control procedure. In Figure 5, cyclin D1 expression in the control group and sham IgG group was undetectable or minimal. In contrast, cyclin D1 expression was clearly observed in anti-HMGB1 antibody-treated animals at 48 h after APAP administration.

Cyclin D1 expression in the sham and anti-HMGB1 groups was the same level as in the control group that was almost undetectable at 24 h time point.

### Hepatic BrdU expression

**Figure 6:**
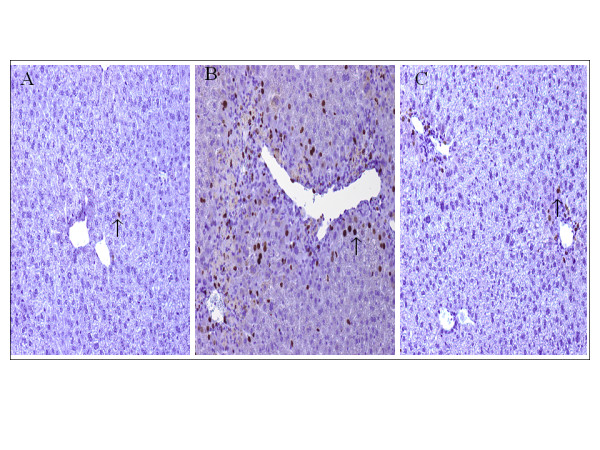
**Effect of treatment with sham or anti-HMGB1 antibodies on hepatocyte regeneration in acetaminophen (APAP)-injected mice.** 5-bromo-2-deoxyuridine (BrdU) staining was assessed at 48 hours after induction of acute liver injury (ALI) (or sham procedure, n = 6 for each group). A typical picture is shown. BrdU-positive nuclei are indicated by arrows. (A = 48 h Control, B = 48 h Sham IgG, C = 48 h anti-HMGB1).

The hepatocyte proliferation was assessed by BrdU immunohistological staining. BrdU-positive nuclei were shown by the arrows. At 48 hours, the number of labeled nuclei (per low power) was significantly increased in both sham IgG (71 ± 8) (Figure 6B) and anti-HMGB1 (16 ± 4) (Figure 6C) groups, although to a statistically lesser extent in the anti-HMGB1 treated mice; after 48 h, the extent of hepatic BrdU expression, however, depended mainly on the extent of damage, because it was significantly correlated with the area of hepatocyte necrosis for each mouse [17]; our result showed that the sham IgG group had larger necrotic area (14.6 ± 1.2%) than the anti-HMGB1 group (1.6 ± 0.3%), resultantly, the sham IgG group had a larger number of BrdU-positive cells than the anti-HMGB1 group, our result was consistent with Javier Vaquero’s report [17].

## Discussion

HMGB1 is a well-known and studied protein, which is very conservative among species and acts as a nuclear protein that promotes transcriptional activation. It acts late in the time line as a downstream inflammatory mediator in sepsis [10]. HMGB1 also is released readily from necrotic or damaged cells, and serves as a signal for inflammation [19]. The purpose of this study was to test the notion that HMGB1 impairs hepatocyte regeneration after APAP overdose. The major and the novel findings of this investigation are: (a) serum HMGB1 is increased after APAP challenge and anti-HMGB1 therapy improves hepatocyte regeneration seen in pathology 24 hours after APAP administration; (b) blockade of HMGB1 decreases serum ALT /AST and enhances liver recovery at 48 h time point (c) the late phase improvement is associated with an augmented NF-κB DNA binding (d) blockade of HMGB1 significantly increases the expression of cell cycle protein cyclin D1 in liver tissue at 48 h time point.

In this study, the ALT/AST concentrations were very high at 24 h time point, however, those surviving mice with high ALT/AST levels did not look severely ill, most of them looked only mildly distressed. In a pilot experiment with a lower dose of APAP (250 mg/kg) administration, the ALT level could rapidly decrease to about 100 u/L at 48 h, however, the pathology at 48 h looked much worse than the liver injuries with similar ALT/AST levels caused by hemorrhagic shock and sepsis, therefore, we think ALT/AST concentrations alone can not accurately predict the severity of APAP hepatotoxicity.

In this study, ELISA was also performed to measure 24 h serum HMGB1 level by using HMGB1 ELISA kits from Shino-Test (Kanagawa, Japan). The serum HMGB1 concentrations were 2 ± 0.2 ng/mL in the normal control group and 23 ± 1.4 ng /mL in APAP injected animals (n = 5 for each group, data presented as mean ± SEM), however, our results in the APAP group were 12 times lower than Antoine DJ’ report [20] who used HMGB1 neutralizing antibody from Shino-Test Corporation (Tokyo, Japan), this big difference between the two reports is probably due to the unconvincing HMGB1 ELISA kits, because the manufacturer requires that the serum should be incubated for 24 hours at 37°C, this key step is unusual and rationally unconvincing, this might make the HMGB1 ELISA procedure unreliable and Shino-Test HMGB1 ELISA kits (Kanagawa, Japan) is currently the only available commercial HMGB1 ELISA kits; in contrast, HMGB1 western blot is the conventional method and is still commonly used, currently the HMGB1 western blot is probably more reliable than HMGB1 ELISA procedure, therefore, HMGB1 western blot is shown in this study.

Antoine DJ et al [20] showed that blockade of HMGB1 significantly decreased serum ALT/AST and injury score at 24 h time point, this is different from our 24 h results in which neutralization of HMGB1 did not significantly decrease serum ALT/AST but enhanced hepatocyte regeneration in HE staining, our 24 h results suggest that HMGB1 probably is not an important early injurious factor in this model, this notion is further supported by our previous study [11] in which a high dose of exogenous HMGB1 injection increased ALT level to 350 u/L in normal mice at 18 h time point; in contrast, APAP overdose could increase ALT level to 4000–10000 u/L at 24 h in mice. Blockade of HMGB1 from two different research groups had different results at 24 h time point, suggest that the role of HMGB1 in this model is still controversial and more investigation is needed. The different 24 h results from two different research groups are probably caused by different antibody sensitivity and specificity. Our study also showed that HMGB1 was involved in liver regeneration, in which the later time point is important, however, the later time point or underlying mechanisms were not studied in Antoine DJ’s report [20].

To elucidate the molecular basis of liver recovery in blocking of HMGB1, we investigated its effect on NF-κB signaling pathway because currently, NF- κB is thought to play a major role in the initiation of liver regeneration after cell or tissue loss (such as partial hepatectomy) [18,21], and enhanced NF-kB DNA binding is associated with better liver recovery in APAP hepatotoxicity [22]. Our data suggested that blockade of HMGB1 is associated with a beneficial response characterized by activation of NF-κB. Although NF-κB activation modulates inflammation [10], it is also known to protect hepatocytes from cell death, and inhibition of NF-κB after partial hepatectomy results in massive hepatocyte apoptosis, worsens liver injury and decreases survival [23]. There is evidence suggesting that the impact of APAP toxicity ensues, at least in part, by dramatic modulation of inflammatory and/ or regeneration programs [24]. It is possible that in HMGB1–blocked mice subjected to APAP overdose, enhanced NF-κB activation diverts intracellular pathways from those associated with inflammation and cell death, to mechanisms linked to recruitment and activation of pro-regenerative programs, therefore, activation of NF-κB by blockade of HMGB1 might facilitate regeneration in this ALI induced by APAP.

Liver regeneration is a vital process for survival after a toxic insult [25-27]. Regeneration ensures the replacement of necrotic cells and the full recovery of organ function. The exposure to growth factor such as hepatocyte growth factor results in the expression of cell cycle proteins [18]. The induction of cyclin D1 is the most reliable marker for cell cycle (G _1_ phase) progression in hepatocytes [18]. Once hepatocytes express cyclin D1, they have passed the G_1_ restriction point and are committed to DNA replication [18]. In current investigation, our western blot data showed that neutralization of HMGB1 markedly increased the level of cyclin D1 in the APAP challenged liver tissue. These changes in cyclin D1 expression were associated with decreased serum ALT /AST and improved liver regeneration in anti-HMGB1 antibody –treated mice receiving APAP, suggesting that blockade of HMGB1 likely facilitates activation of cyclin D1-mediated regeneration pathways, and the increased cyclin D1 expression might be modulated by enhanced NF-κB DNA binding [18]. Our data suggest that HMGB1 is an important factor, which impairs hepatocyte regeneration in APAP overdose. Anti-HMGB1 antibody treatment lowered serum ALT/AST at 48 h time point, probably by blocking HMGB1 to reduce inflammation and to improve hepatocyte regeneration, resultantly hastened liver recovery from APAP hepatotoxicity.

Currently N-acetyl-cysteine (NAC), a glutathione precursor, is the antidote for APAP overdose [28]. However, this antidotal therapy is effective only for patients who present within hours of an acute overdose, and is less effective for late-presenting patients [28,29]. In addition, prolonged treatment with NAC delays liver recovery from APAP hepatotoxicity [30]. Up to date treating delayed cases with APAP overdose is still problematic, additional therapies are needed. Treatment with anti-HMGB1 neutralizing antibody would serve as an adjuvant to NAC therapy. Further experiments designed to compare the two therapies are needed in the future.

## Conclusion

HMGB1 impairs hepatocyte regeneration after APAP overdose; blockade of HMGB1 enhances liver recovery and may present a novel therapy to treat APAP overdose.

## Competing interests

The authors declare that they have no competing interests.

## Authors’ contributions

RKY designed the study. All authors participated in the animal handling and procedures. All authors read and approved the final manuscript.

## Pre-publication history

The pre-publication history for this paper can be accessed here:

http://www.biomedcentral.com/1471-230X/12/45/prepub
